# Substance use and its effect on antiretroviral treatment adherence among male fisherfolk living with HIV/AIDS in Uganda

**DOI:** 10.1371/journal.pone.0216892

**Published:** 2019-06-03

**Authors:** Katelyn M. Sileo, Williams Kizito, Rhoda K. Wanyenze, Harriet Chemusto, Elizabeth Reed, Jamila K. Stockman, William Musoke, Barbara Mukasa, Susan M. Kiene

**Affiliations:** 1 San Diego State University School of Public Health, San Diego, California, United States of America; 2 Mildmay Uganda, Lweza, Kampala, Uganda; 3 Makerere School of Public Health, Makerere University, Kampala, Uganda; 4 Division of Global Public Health in the Department of Medicine, University of California San Diego, La Jolla, California, United States of America; New York Blood Center, UNITED STATES

## Abstract

**Background:**

Fisherfolk are a most-at-risk population for HIV being prioritized for the scale up of HIV treatment in Uganda. Heavy alcohol use and potential drug use may be a major barrier to treatment adherence for men in this setting.

**Objective:**

This study examines the prevalence of substance use, and its influence on antiretroviral treatment (ART) adherence, among male fisherfolk on ART in Wakiso District, Uganda.

**Methods:**

This cross-sectional study included structured questionnaires (N = 300) with men attending HIV clinics near Lake Victoria. Using generalized logistic modeling analyses with a binomial distribution and logit link, we conducted multivariate models to test the association between each alcohol variable (quantity and frequency index, hazardous drinking) and missed pills, adjusting for covariates, and tested for interactions between number of pills prescribed and alcohol variables.

**Results:**

Thirty-one percent of men reported sub-optimal adherence. Half (46.7%) reported drinking, of which 64.8% met criteria for hazardous drinking. Illicit drug use was low (6%). In the multivariate model, men with greater scores on the alcohol frequency and quantity index were more likely to report missed pills compared to those reporting no drinking (AOR: 1.60, 95% CI: 1.29–1.97). Hazardous drinking had a greater effect on missed ARV doses among men taking twice daily regimens compared to once daily (AOR: 4.91, 95% CI: 1.68–14.37).

**Conclusions:**

Our findings highlight the need for targeted alcohol-reduction interventions for male fisherfolk on ART who drink at high quantities to improve ART adherence and to prevent the known negative health effects of alcohol for HIV-infected individuals.

## Introduction

In Uganda and throughout sub-Saharan Africa, national strategies for HIV/AIDS prevention and control are increasingly centered on the scale up of antiretroviral treatment (ART). When taken as prescribed, antiretrovirals (ARVs) have the ability to reduce one’s viral load to non-infectious levels, which extends the life years of people living with HIV (PLHIV) substantially, and has the potential to curb the population-level incidence of HIV [1, 2]. In an effort to achieve universal access to treatment globally, the World Health Organization (WHO) has issued recommendations for the expansion of ART eligibility to all, regardless of CD4 cell count or disease stage [3]. The Ugandan Ministry of Health (MOH) is currently rolling out this policy, which has been in place for most-at-risk populations (MARPs) since 2014 [4]. In Ugandan fishing villages, HIV prevalence is estimated between 20–30% [5–7] compared to 5.9% nationally [8]. Thus, fisherfolk, or individuals living and working in the fishing communities on Lake Victoria, are a MARP being prioritized for test-and-treat initiatives [4, 9]. However, little is known about HIV care engagement with this population.

In order to achieve and sustain viral suppression and realize the full benefits of ART, it is necessary that PLHIV take 95% of their ARVs as prescribed [10]. In sub Saharan Africa, men on ART have worse treatment adherence and greater mortality compared to women [11–13]. Men in fishing communities are likely to encounter even greater barriers to ART adherence than men generally–due to frequent travel, conflicting work responsibilities, distance and other structural barriers to HIV care [14, 15]. Further, high risk alcohol use and drug use observed in this setting [5, 9, 15–18] may pose a significant threat to men’s ART adherence. Substance use among PLHIV is a significant public health concern [19], as it can advance HIV/AIDS disease progression by directly decreasing CD4, and indirectly, through its effect on poor treatment adherence [20–23]. A meta-analysis of 40 studies reported alcohol drinkers were approximately 50–60% less likely to be classified as adherent, compared to those who abstained or drank relatively less [24].

Tumwesigye et al. [16] found 69.8% of fishermen drank at hazardous levels in two Ugandan fishing communities. Moreover, hazardous alcohol use, defined as a pattern of alcohol consumption that increases the risk of harmful consequences for the user and others [25], has been described in qualitative studies as part of the broader culture of fishermen [14]. Other drug use, including marijuana and khat (a plant-based stimulant) may also be high among fisherfolk [9, 18]; however, more data is needed on the prevalence of drug use in this setting. While alcohol use is reportedly high among fishing communities [5, 9, 15–18], and studies with fishermen identify alcohol use as a risk factor for HIV acquisition [5, 9, 18, 26] and a major barrier to prevention services and linkage to HIV care [27, 28], to our knowledge no research has examined the level and patterns of alcohol and drug use among fisherfolk living with HIV and on ART. Substance use may differ among PLHIV compared to the general population; some studies in Uganda report high rates of alcohol use and misuse among PLHIV [29], and others report temporary reductions in unhealthy drinking upon ART initiation, but a return to baseline drinking over time [30].

In this cross-sectional study, we examine the prevalence of substance use, as well as the role of substance use in HIV medication adherence among male fisherfolk on ART in Wakiso District, Uganda. We focused specifically on fisherfolk because we hypothesized that they would have unique barriers to HIV care engagement, including hazardous drinking, and we included only men based on evidence of a gender gap disadvantaging men in the HIV care continuum [11–13]. We test the associations between substance use and ART adherence, hypothesizing that those who drink and/or use drugs will have worse adherence. We also explore which risk levels of drinking may drive associations between alcohol use and adherence, and based on findings from prior studies [24, 31], explore the potential modifying effect of number of ARV pills prescribed on this relationship. This paper is the first to examine the prevalence of substance use, including alcohol and other drug use, and its effect on treatment adherence among Ugandan fisherfolk on ART. With fisherfolk a priority population for test-and-treat initiatives in Uganda and a key population in other African settings, research on substance use and treatment adherence could have important implications for the development of interventions to improve engagement in HIV care through substance use reduction for this most-at-risk population.

## Methods

This cross-sectional study included a quantitative assessment conducted between October 2016 and March 2017 at seven health centers/outreach sites in Wakiso District, Uganda. Sites included three on land (Kasenyi outreach, Entebbe Hospital, Kigungu Health Centre III) and four on islands (Rapha Health Centre [Bussi Island], ZZinga outreach [Zzinga Island], Bussi Health Centre [Bussi Island], Kachanga outreach [Kachanga Island]). Rapha Health Centre is a private not-for-profit facilty; Bussi HCIII, Kigungu, and Entebbe Hospital are governmental public health facilities. These clinics were purposively sampled because they are near fishing communities and therefore serve large populations of fisherfolk. Kasenyi, Kachanga, and Zzinga are outreach sites, which serve the surrounding landing sites and islands. We worked in collaboration with Mildmay Uganda in the implementation of the study, a non-governmental organization that partners with the Ugandan MOH to provide free HIV services to patients, including HIV testing, care, and treatment. Eligibility for ARVs at the time of the study was CD4<500 in the broader population in Uganda, but among fisherfolk and other MARPs, the MOH and Mildmay Uganda were implementing WHO recommendations for immediate initiation of ART upon diagnosis regardless of CD4 cell count or WHO clinical stage; in practice, this included same day initiation, except for men testing in community settings who initiated after linking to an HIV clinic.

The research population included male fisherfolk living with HIV/AIDS, including fishermen and other occupations supporting the fishing industry (fish sellers, boat loaders/operators). Our additional eligibility criteria were: aged 18 years or older and prescribed ART for at least 6 months (to measure adherence). Using purposive sampling, clinic staff and a trained research assistant recruited men for participation as they presented to the HIV clinic/outreach clinics during routine HIV appointments. Study staff non-systematically approached men to inform them about the study and assess their eligibility. We also invited men to participate during routine reminder calls for HIV clinic appointments. During calls, the research assistant informed men of the opportunity to participate in the study at their next appointment or to schedule an appointment at a time more convenient to them. A total of 64 men were recruited via telephone and participated at a future clinic appointment. Of the 322 eligible men invited to participate, 22 declined for the following reasons: did not have time (n = 14), did not want to disclose sensitive information (n = 4), intoxicated (n = 1), hearing impairment (n = 1), wanted more compensation (n = 1), wanted to be interviewed with wife (n = 1). No additional data was collected from these men; therefore, we are unable to assess differences between eligible men who accepted vs. declined participation in this study.

Participants meeting eligibility criteria and providing written informed consent completed an interviewer-administered questionnaire immediately after enrolling in the study. The research assistant conducted the individual structured computer-based interviews (<45 minutes duration) in a private space in the HIV clinic or another agreed upon location. Participants received 10,000 Ugandan Shillings (~2.75 USD) to compensate them for their time in the structured interview. All procedures were approved by the institutional review boards at San Diego State University and Makerere University School of Public Health, as well as the Uganda National Council for Science and Technology.

### Measures

#### Covariates

Among the socio-demographics and HIV-related variables measured, we include the following variables as potential covariates: age, education, monthly income, occupation, marital/partner status, research site, mobility, time spent traveling to the HIV clinic, time since HIV diagnosis, time since ART initiation, and number of pills prescribed per day. Based on the distribution of the data, several variables were dichotomized for analysis, including education (no schooling vs. any schooling), employment status (fishermen vs. other fishing occupations), marital status (married vs. not married/separated), research site (landing site vs. island), mobility (traveled/slept outside of the community in the prior year, yes or no), and number of pills prescribed per day (once daily vs. twice daily). Continuous variables included monthly income (1 unit equates to 50,000 shillings), time spent traveling to the HIV clinic (1 units equates to 30 minutes), and time on ART and since diagnosis (1 unit equates to 6 months).

#### Alcohol variables

We used the WHO’s Alcohol Use Disorders Identification Test (AUDIT) to assess drinking risk levels [32] (present sample, α = 0.63). The AUDIT includes cut-off scores to categorize individuals at different levels of risky drinking, which we use for data analysis, including: hazardous alcohol use (score of 8 or more), high risk or harmful use (16–19), and dependence (20 or more) [25]. By taking the product of the first two AUDIT items assessing frequency and quantity of alcohol use, we also calculated an index of current drinking frequency and quantity; this measure weighs the quantity of alcohol typically consumed by frequency of use and is a more proximal measure to current drinking than the AUDIT risk categories, which asks about the prior year. In addition, we assessed the type of alcohol typically consumed. Response options included alcoholic beverages common in the study setting: bottled beer (4.5–7% alcohol by volume [ABV]), tonto (locally brewed fermented banana juice, 3% ABV), malwa (locally brewed from millet, communally shared in single container with straws, 3% ABV), Waragi (traditional gin, 48.4% ABV), “other kind of local brew,” and “other kind of alcohol.”

#### Other drug use variables

Drug use was assessed by the National Institute of Drug Abuse (NIDA) Modified ASSIST scale [33], adapted for the Ugandan context. Participants were asked about their lifetime use of substances, use within the prior 3 months, and the extent of use for each type of drug reported in the prior 3 months. We included all drugs in the original NIDA ASSIST, adapted to include the Lugandan (local language) and local names for each. Drugs included: cannabis, cocaine, prescription stimulants, methamphetamine, inhalants, sedatives or sleeping pills, hallucinogens, street opioids, prescription opioids, and tobacco. We added substances specific to Uganda, derived from consultation with Ugandan experts in drug use, and the piloting of items locally, including: kuber (a plant-based stimulant, chewed or consumed as a tea), shisha (a plant-based stimulant, commonly smoked with marijuana), and khat (a plant-based stimulant, chewed).

#### Adherence outcome

The Adult AIDS Clinical Trial Group (AACTG) scale was used to measure self-reported ARV adherence [34]. The AACTG includes recall questions about ARVs missed for the previous four days prior to the interview and items measuring reasons for non-adherence. It has demonstrated construct validity in Uganda and similar settings [35]. For analysis, we assess the proportion of missed ARVs in the prior four days, out of total ARVs prescribed. In addition to self-reported adherence, we documented initiation date, and ART regimen from participants’ medical records. See S1 File for a copy of the study measures.

#### Data analysis plan

In SPSS version 24, we characterize the prevalence of alcohol and other substance use using descriptive statistics. We identified outliers (values greater than 3 standard deviations [SD] outside of the mean) [36] and truncated them to the highest value within 3 SDs of the mean for the following variables: monthly income, months since diagnosis, months on ART, and minutes traveled to the clinic. We used generalized logistic modeling analyses with a binomial distribution and logit link with our outcome in the form of events within trials, with events being the number of missed ARVs and trials being the number of ARVs prescribed over a four-day recall period (possible range given regimens prescribed was 4–8). We tested bivariate associations between potential independent variables, including socio-demographic and HIV variables (age, education, monthly income, occupation, marital/partner status, research site, mobility, time spent traveling to the HIV clinic, time since HIV diagnosis, time since ART initiation, number of pills prescribed) and missed pills, to determine which variables to include in the final models. We also planned to test the bivariate associations between the quantity and frequency index and drinking by AUDIT risk categories (risky drinking, hazardous, dependent) with missed pills, contingent on prevalence. Finally, we tested separate multivariate models for each alcohol variable (AUDIT risk categories, quantity and frequency index) associated with missed pills (p < 0.10 in bivariate models), adjusting for statistically significant covariates (p < 0.10), and testing for interactions between number of pills prescribed and alcohol variables. Odds Ratios (OR) and Adjusted Odds Ratios (AOR) from bivariate and multivariate analysis with 95% confidence intervals are presented. In multivariate models, we drop covariates that lost statistical significance (p < 0.10), presenting only the trimmed models. We planned to mirror the analyses described for alcohol, with the drug use variables, dependent on sufficient prevalence among the sample.

## Results

### Participant characteristics

The average age of our full sample (N = 300) was 37 (SD = 8.6), and most men reported being employed as a fisherman (82%) and never attending school (67.3%). On average, men had been aware of their status for about two years (mean = 24.6 months, SD = 17.8) and were initiated on ART soon after diagnosis (mean = 22.9 months, SD = 13.5). Men reported taking 87.8% (SD = 21.5%) of their ARV pills over the 4-day recall period, making 69% of the sample adherent (using a criterion for optimal adherence of 95% of pills taken as prescribed), and about a third of the sample was sub-optimally adherent (31%). See Table 1 for more details on sample characteristics.

**Table 1:** Participant characteristics, alcohol and other substance use, Uganda 2016–2017, N = 300.

	n (%)	Mean	SD	Range
**Demographics**				
Age		36.9	8.6	20–70
Education				
No schooling	202 (67.3%)			
Primary level	84 (28.0%)			
Secondary level	14 (4.7%)			
Marital status				
Never married	30 (10.0%)			
Divorced	78 (26.0%)			
Widowed	16 (5.3%)			
Married & separated most of the time	29 (9.7%)			
Married & living together most of the time	147 (49.0%)			
Monthly income (converted to USD)		62.2	29.7	4.1–160.4
Occupation				
Fishermen	246 (82.0%)			
Fish seller, cleaner, dryer	35 (11.7%)			
Boat operator, repairer, loader, other	19 (9.7%)			
Mobility (frequency of travel in prior 12 months)				
No travel	106 (35.3%)			
Once	73 (24.3%)			
2–5 times	91 (30.3%)			
6 or more times	30 (10.0%)			
Travel time to clinic (minutes)		41.4	39.56	2–180
**HIV/AIDS variables**				
Months since HIV diagnosis		24.6	17.8	4–102
Months since ART initiation		22.9	13.5	2–55
Treatment regimen*				
First line regimen	298 (99.3%)			
TDF+3TC+EFV (Preferred)	260 (87.2%)			
AZT+3TC+EFV	11 (3.7%)			
AZT+3TC+NVP	24 (8.0%)			
TDF+3TC+NVP	3 (1.0%)			
Second line regimen	2 (0.7%)			
TDF/3TC	1 (50.0%)			
AZT+3TC	1 (50.0%)			
Number of pills prescribed per day				
Once daily dosing	260 (86.7%)			
Twice daily dosing	40 (13.3%)			
ART Adherence (% of prescribed pills taken)		87.8%	21.5%	0–100%
Sub-optimal adherence (< 95% of pills taken as prescribed)				
Yes	93 (31.0%)			
No	207 (69.0%)			
**Alcohol use**				
Type of alcohol typically consumed*				
Ttonto	43 (30.3%)			
Waragi	35 (24.6%)			
Malwa	28 (19.7%)			
Bottled beer	26 (18.3%)			
Any other kind of local brew	8 (5.6%)			
Any other kind of alcohol	1 (1.4%)			
Alcohol quantity (drinkers only)*		2.92	1.54	1–10
AUDIT score		4.5	5.1	0–20
AUDIT score (drinkers only)*		9.0	3.9	2–20
Hazardous alcohol use (AUDIT = /> 8)	92 (30.7%)			
Hazardous alcohol use (AUDIT = /> 8) (drinkers only)*	92 (64.8%)			
High risk alcohol use (AUDIT = /> 16)	8 (2.7%)			
Alcohol dependence (AUDIT = />20)	1 (0.30%)			
**Drug use (excluding alcohol)**				
Prescription drug use for non-medical reasons (ever)				
Yes	9 (3.0%)			
No	291 (97.0%)			
Illicit drug use (ever)				
Yes	18 (6.0%)			
No	282 (94.0%)			
Drugs used (ever)				
Cannabis	2 (0.70%)			
Tobacco	15 (5.0%)			
Kuber (stimulant)	7 (2.3%)			
Sisha (stimulant)	5 (1.7%)			
Khat (stimulant)	3 (1.0%)			

Note: Adherence calculated based on the proportion of pills taken over a four day recall period of total pills prescribed, based on the Adult AIDS Clinical Trial Group (AACTG) scale (Chesney et al., 2000);

* indicates items assessed only among participants reporting alcohol consumption in the prior 12 months (n = 142);

alcohol quantity = number of drinks typically consumed on drinking days; AUDIT = Alcohol Use Disorder Identification Test (World Health Organization, 2001); variables calculated from AUDIT score assess alcohol use in the prior year; Ttonto is a locally brewed fermented banana juice, 3% volume/volume percent (v/v), malwa is locally brewed from finger millet, communally shared in single container with straws, 3% v/v; Waragi is traditional gin, 48.4% v/v.

### Prevalence of alcohol use

Approximately half of men reported drinking in the prior year (47.3%) as well as in the prior 30 days (46.7%). Tonto (30.3%) and Waragi (24.6%) were the most commonly consumed drinks. Men reported an average of almost 3 drinks per drinking day for beverages other than malwa. Among those reporting any alcohol use, 40% of those who drink reported ever binge drinking, though infrequently (2.1% at least once/week, 9.2% at least once/month, 23.9% less than once/month). Among men reporting any drinking, the number of drinks typically consumed on drinking days was 2.92 (SD = 1.54, range 1–10), which we adjusted based on the alcohol content and serving size of the type of alcohol they reported most typically consuming [37], with the exception of malwa which does not come in a standardized container and is difficult to measure consumption volume because it is consumed communally. The average AUDIT score was 4.5 (SD = 5.13), and nearly a third (30.7%) of the sample met criterion for hazardous drinking (AUDIT score ≥ 8) in the prior year; among those reporting any drinking, two-thirds drank at hazardous levels (64.8%). Only eight men met AUDIT criterion of high-risk drinking (AUDIT ≥ 16), and one for alcohol dependence (AUDIT ≥ 20). Descriptive statistics for alcohol use are displayed in Table 1.

### Prevalence of drug use, excluding alcohol

Only 6% of the sample reported ever using drugs other than alcohol, and only 3% reported using prescription drugs for non-medical reasons. Among the 18 men who reported any drug use other than alcohol, illicit substances included kuber (n = 7), sisha (n = 5), khat (n = 3), and Cannabis (n = 2); tobacco use was also reported (n = 15). Table 1 includes descriptive statistics for drug use. Due to the low use of other drugs, we focus the remainder of the findings on alcohol use.

### Effect of alcohol use on ART adherence

In bivariate and multivariate analyses, we operationalized alcohol use in two ways: alcohol use quantity/frequency index and hazardous alcohol use (AUDIT ≥ 8) (yes or no). For hazardous alcohol use, we pooled no drinking and low risk drinking together as the reference group, as the mean adherence percentage demonstrated no difference in adherence levels between no drinking and low risk drinking (AUDIT = 1–7) (mean adherence for non-drinkers = 88.49%, for low risk drinkers = 88.70%). In addition, we did not separate the AUDIT categories of high-risk/harmful drinking (AUDIT ≥ 16) or alcohol dependence (AUDIT ≥ 20) for analysis due to their low prevalence in the prior year according to the AUDIT.

In unadjusted models, greater scores on the alcohol use quantity and frequency index was positively associated with greater odds of reporting missed ARVs (OR: 1.56, 95% CI: 1.27–1.92, p < 0.001). Compared to no drinking or low risk drinking (AUDIT = 1–7), men meeting criteria for hazardous alcohol use (AUDIT ≥ 8) had a 1.39 (95% CI: 0.98–1.98) greater odds of non-adherence; however, the effect was marginally significant (p = 0.06). Covariates found statistically associated with missed pills (p < 0.10) in bivariate models included: younger age, greater education, lower monthly income, mobility in the prior 12 months, greater travel time to HIV clinic, and being prescribed a once daily (ART) regimen (compared to twice daily). See Table 2 for detailed statistics.

**Table 2:** Bivariate associations between independent variables and missed antiretroviral medication, Uganda 2016–17, N = 300.

	OR (95% CI)	x^2^	p
**Demographics and HIV variables**			
Age	0.96 (0.94–0.98)	12.99	**<0.001**
Education			
Any schooling	1.58 (1.12–2.22)	6.75	**0.01**
No schooling (ref)			
Marital status			
Not married or separated	1.05 (0.75–1.46)	0.07	0.79
Married (ref)			
Research site			
Landing site	0.92 (0.66–1.30)	0.21	0.65
Island (ref)			
Monthly income	0.92 (0.84–1.00)	3.86	0.05^†^
Occupation			
Fishermen	1.03 (0.67–1.56)	0.01	0.91
Other fishing occupation (ref)			
Mobility			
Yes	1.65 (1.14–2.40)	6.92	**0.01**
No (ref)			
Travel time to clinic	1.13 (1.01–1.26)	4.33	**0.04**
Months since HIV diagnosis	0.99 (0.94–1.04)	0.86	0.77
Months since ART initiation	0.93 (0.87–1.00)	3.69	0.06^†^
Number of pills prescribed per day			
Twice daily dosing	0.37 (0.22–0.62)	14.28	**<0.001**
Once daily dosing (ref)			
**Alcohol variables**			
Alcohol use quantity and frequency index	1.56 (1.27–1.92)	18.29	**<0.001**
Hazardous alcohol use			
Yes (AUDIT = /> 8)	1.39 (0.98–1.98)	3.43	0.06^†^
No (no drinking and AUDIT <8) (ref)			

Note:

Bold text indicates associations p < .05;

^†^p < .10;

OR: Odds Ratio;

x^2^ = Wald chi square;

AUDIT = Alcohol Use Disorder Identification Test; ART = antiretroviral treatment; ref = reference group; Monthly income: 1 unit = 50,000 UGX; Travel time to clinic: 1 unit = 30 minutes; Months since HIV diagnosis: 1 unit = 6 months; Months since ART initiation: 1 unit = 6 months.

In the final trimmed model testing the association between the alcohol use quantity and frequency index and missed ARVs adjusted for covariates (see Table 3), a positive main effect remained, with greater scores on the alcohol use quantity and frequency index positively associated with greater odds of reporting missed ARVs (AOR: 1.60, 95% CI: 1.29–1.97, p < 0.001). Negative main effects for younger age and number of pills prescribed also remained in the final model, with decreased odds of missed ARVs greater among older men (AOR: 0.97, 95% CI: 0.94–0.99, p < 0.001) and men taking twice daily regimens (compared to once daily) (AOR: 0.43, 95% CI: 0.25–0.74, p = 0.02). We also found men with any education had greater likelihood of missed ARVs compared to no education (AOR: 1.51, 95% CI: 1.06–2.14, p = 0.02). A marginally significant negative association remained for monthly income (AOR: 0.73, 95% CI: 0.51–1.04, p = 0.09). No statistically significant interaction was identified between the alcohol index and number of pills prescribed.

**Table 3:** Multivariate models testing associations between alcohol use quantity and frequency index and missed antiretroviral medication, Uganda 2016–17, N = 300.

	AOR (95% CI)	x^2^	p
Age	0.97 (0.94–0.99)	7.03	**<0.001**
Education			
Any schooling	1.51 (1.06–2.14)	5.24	**0.02**
No schooling (ref)			
Monthly income	0.73 (0.51–1.04)	2.97	0.09^†^
Number of pills			
Twice daily dosing	0.43 (0.25–0.74)	9.38	**0.02**
Once daily dosing (ref)			
Alcohol use quantity and frequency index	1.60 (1.29–1.97)	18.97	**<0.001**

Bold text indicates associations p < .05;

^†^p < .10;

AOR: Adjusted Odds Ratio;

x^2^ = Wald chi square;

ref = reference group.

**Fig 1:**
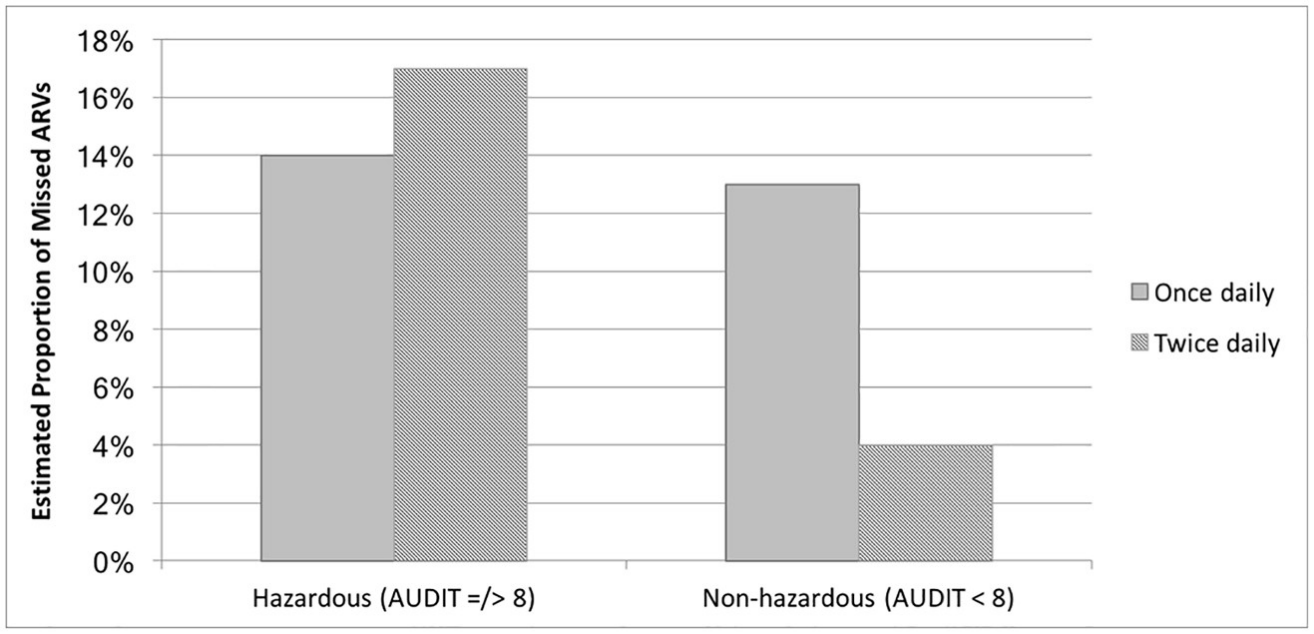
Interaction between any hazardous alcohol use and pills prescribed per day on the proportion of missed pills over a 4-day recall period, controlling for covariates. Men taking twice daily dosing and reporting hazardous alcohol use were nearly five times as likely to report missed antiretrovirals (ARVs) than men taking twice daily dosing but reporting no hazardous alcohol use (AOR: 4.91, 95% CI: 1.68–14.37, p = 0.004). Covariates were fixed at their mean value. Full statistics reported in Table 4.

Table 4 displays the final trimmed multivariate model testing the association between hazardous drinking and ART adherence, adjusted for covariates. The main effect between hazardous drinking and missed ARVs was not statistically significant in the full model (AOR: 1.10, 95% CI: 0.75–1.61, p = 0.63). Younger age (AOR: 0.96, 95% CI: 0.94–0.99, p = 0.002), any education compared to no education (AOR: 1.54, 95% CI: 1.08–2.18, p = 0.02), and taking a twice-daily regimen compared to once daily (AOR: 0.26, 0.12–0.55, p < 0.001) remained significantly associated with missed pills in the full model. There was a statistically significant interaction between hazardous alcohol consumption and number of pills prescribed; men taking twice daily dosing and reporting hazardous alcohol use were nearly five times as likely to report missed ARVs than men taking twice daily dosing but reporting no hazardous alcohol use (AOR: 4.91, 95% CI: 1.68–14.37, p = 0.004); see Table 4 for statistics and Fig 1 for a graphic depiction of the observed interaction.

**Table 4:** Multivariate models testing associations between hazardous drinking and missed antiretroviral medication, Uganda 2016–17, N = 300.

	AOR (95% CI)	x^2^	p
Age	0.96 (0.94–0.99)	9.68	**0.002**
Education			
Any schooling	1.54 (1.08–2.18)	5.75	**0.02**
No schooling (ref)			
Number of pills			
Twice daily dosing	0.26 (0.12–0.55)	12.66	**<0.001**
Once daily dosing (ref)			
Any hazardous drinking			
Yes	1.10 (0.75–1.61)	0.23	0.63
No (ref)			
Hazardous drinking x Number of pills	4.91 (1.68–14.37)	1.68	**0.004**

Note:

Bold text indicates associations p < .05;

AOR: Adjusted Odds Ratio;

x^2^ = Wald chi square;

Risky drinking = Score of 8 or greater on the Alcohol Use Disorder Identification Test (AUDIT); hazardous alcohol use is defined as AUDIT score equal or greater to 8 and no hazardous alcohol use defined as AUDIT score less than 8; ref = reference group.

## Discussion

Despite PLHIV in fishing communities being prioritized for the scale up of test-and-treat initiatives in Uganda, little is known about substance use in this population or its effect on optimal ART adherence; this is the first study to our knowledge to begin to fill this gap in the literature. Among our sample of men enrolled in HIV care, approximately a third reported sub-optimal ART adherence (< 95% of ARVs taken as prescribed). Though half the sample reported abstaining from alcohol use altogether, a third of men met criteria for “hazardous” alcohol use, highlighting the need for alcohol intervention with a portion of men. This is especially needed given the finding that alcohol use was associated with an increased likelihood of missed ARVs among men–with the greatest effect on men on a twice-daily ART regimen.

Our findings add to the broader literature [24, 38] demonstrating greater alcohol consumption is associated with greater likelihood of missed ARVs. We also found men reporting no drinking and men reporting low risk drinking had similar rates of adherence, suggesting the consumption of higher quantities of alcohol may be driving the alcohol-adherence association. However, no direct association was found for hazardous drinking (which encompasses other negative outcomes of drinking in addition to quantity) and missed ARVs. In the literature, there have been mixed findings on the role of alcohol quantity and risk levels on adherence; some studies suggest any drinking regardless of amount or low risk drinking may drive the alcohol-adherence association [31, 38–42], while others suggest higher quantities of drinking drive this association [24, 38, 43]. Though our findings support the latter, more research is needed to better understand the relationship between alcohol use and non-adherence.

Counter to evidence on the advantage of once-daily single tablet regimens for treatment adherence [44], we found a negative direct effect between number of pills prescribed (i.e., once daily vs. twice daily regimens) and adherence; that is, men prescribed once daily regimens reported overall worse adherence than men taking twice daily regimens. However, as demonstrated by the observed interaction between hazardous drinking and number of pills prescribed (see Fig 1), for men classified as hazardous drinkers, those on twice daily dosing had worse adherence compared to those on once daily regimens. One possible explanation that men on twice daily regimens had better adherence overall may be that they were receiving more intensive ART adherence counseling and monitoring since treatment failure on the once daily regimen. Further, men taking twice daily regimens who drink at hazardous levels may have failed on the preferred first line regimen because of alcohol use. For these men, increased adherence counseling and monitoring, which was shown to be working for all other men, may not be addressing their main barrier to adherence–alcohol use. Being switched to twice daily dosing despite increased monitoring may especially increase risk of non-adherence among hazardous drinkers, who may have more difficulty remembering or planning their alcohol consumption around two pills compared to one. Prior studies similarly report regimen complexity is an important factor in alcohol’s influence on adherence [24, 31], reinforcing the benefits of a once daily regimen for men who drink.

Alcohol consumption levels were lower in our sample of men living with HIV compared to prior studies with non-HIV positive fisherfolk [9, 16]. In addition, while several studies have suggested marijuana and khat may be prevalent in these communities [9, 18], very few men reported drug use. While this in part could be explained by underreporting, a qualitative exploration of changes in men’s drinking since diagnosis (reported elsewhere) revealed men in our sample had made significant efforts to reduce their consumption since diagnosis [45]. Yet hazardous drinking was prevalent among those who do drink; one-third of the overall sample, and two-third of those who drink, were classified as hazardous drinkers on the AUDIT. In addition to the demonstrated negative effects of alcohol on adherence, alcohol’s direct effect on CD4 cell count, and disease progression is dangerous for PLHIV [20, 21, 46], making the level of drinking observed in our sample concerning.

These findings have important implications for clinical practice. First, the integration of screening and brief intervention (SBI) [47] into HIV clinical care with this population may be beneficial, given the observed rates of alcohol consumption and associations between drinking alcohol use and nonadherence. SBI includes universal screening for alcohol misuse, followed by targeted, low-resource, and time-limited (1–2 meetings) efforts to provide information, increase motivation, and teach behavioral change skills to reduce alcohol use [47]. Our findings suggest screening questions should assess both the quantity of alcohol consumed and risk levels, and intervention efforts focused on those screening for higher levels of alcohol use, as opposed to those drinking at lower risk levels, may be most effective. While SBI has gained considerable support in developed settings [48, 49], among the more limited number of studies assessing SBI in sub-Saharan Africa, there is mixed support [50–55]. The successful implementation of SBI and other integrated alcohol-adherence interventions would need to be tailored to the unique context of fishing villages, especially considering significant occupational and structural barriers to HIV care in fishing villages.

The cross-sectional design of our study limits inference of a causal relationship between alcohol and adherence; daily process or event sampling studies are needed to temporally link alcohol-adherence, and quantitatively assess potential mechanisms between the two. Approximately half of our sample reported abstaining from alcohol use and very few men met criteria for harmful drinking/dependence; social desirability may have led to under-reporting of both alcohol use and missed ARVs. The inclusion of biological outcome measures for alcohol use and adherence would have strengthened this study, which relied on self-reported measures. Furthermore, a one-year recall period for the AUDIT may have weakened the observed associations between hazardous drinking and adherence, measured with a 4-day recall period; though our quantity and frequency index provided a measure of current alcohol use. Our alcohol measures also required men to estimate the number of drinks they typically consume. While the interviewer explained to participants the typical size of each type of alcohol assessed during the structured interview, men’s accuracy in this assessment may have varied. In addition, we did not use random sampling to recruit participants; therefore, we cannot generalize our findings to the broader population. Though we did capture men struggling with treatment adherence, by recruiting men directly from the clinic, our sample may over-represent men engaged in HIV care and with better ART adherence.

## Conclusions

As global strategies to end the HIV/AIDS epidemic move towards universal testing and treatment for all [3, 56], efforts to optimize ART adherence, especially with high prevalence populations facing significant barriers to HIV care, are needed. Our findings highlight the need for alcohol-reduction interventions for male fisherfolk on ART in order to improve ART adherence and to prevent the known negative health effects of alcohol for PLHIV [46]. The integration of SBI for alcohol reduction into HIV clinical care may be an effective approach to reduce alcohol consumption and improve adherence among men on treatment who drink. Focusing alcohol reduction and adherence intervention efforts on men reporting higher quantities of alcohol consumption may optimize SBI with this population, and alcohol SBI is especially needed for men on twice daily regimens. Future research is needed to understand how to best tailor and implement an alcohol-reduction intervention given the structural challenges for men in the fishing occupation.

## Supporting information

S1 File Study measures. This questionnaire was administered by an interviewer via Computer-assisted personal interviewing (CAPI) software.
(PDF)

S2 File Study data. This file includes the underlying data for the analyses reported. Variable names correspond with the study measures provided in S1 File. Some information has been removed to de-identify the data and ensure participant confidentiality.
(CSV)
