# Supplementary material for: Substance use and its effect on antiretroviral treatment adherence among male fisherfolk living with HIV/AIDS in Uganda
Source: PLoS One. 2019 Jun 3;14(6):e0216892. doi: 10.1371/journal.pone.0216892 (PMC6546219; doi:10.1371/journal.pone.0216892)
Supplement: S1 File — This questionnaire was administered by an interviewer via Computer-assisted personal interviewing (CAPI) software. (PDF) [file pone.0216892.s001.pdf]

Engagement in Care Questionnaire

Construct	Source
Demographics	Constructed, used in prior studies in Wakiso District landing sites, Uganda
Mobility	Kwena, Z. A., C. S. Camlin, C. A. Shisanya, I. Mwanzo and E. A. Bukusi (2013). "Short-term mobility and the risk of HIV infection among married couples in the fishing communities along Lake Victoria, Kenya." PLoS One 8(1): e54523.
HIV Clinic Attendance, CD4 testing	Constructed, used in prior studies at Gombe Hospital in Butambala District, Uganda
ARV Adherence	**AACTG Adherence Instruments**<br>Chesney MA, Ickovics JR, Chambers DB, et al. Self-reported adherence to antiretroviral medications among participants in HIV clinical trials: The AACTG Adherence Instruments. *AIDS Care.* 2000/06/01 2000;12(3):255-266.
Alcohol Use	**AUDIT,** used in prior studies in Wakiso District landing sites, Uganda<br>WHO (2001). The Alcohol Use Disorders Identification Test: Guidelines for Use in Primary Care. Geneva: WHO.<br><br>**Alcohol Use Disorder DSM-V**<br>The American Psychiatric Association (APA). The Diagnostic and Statistical Manual of Mental Disorders (DSM–5). http://pubs.niaaa.nih.gov/publications/dsmfactsheet/dsmfact.pdf
Drug use	**NIDA Modified ASSIST**<br>National Institute on Drug Abuse (NIDA). NIDA-Modified ASSIST V2.0.<br>https://www.drugabuse.gov/sites/default/files/files/QuickScreen_Updated_2013(1).pdf.

ENGAGEMENT IN HIV CARE AMONG FISHERFOLK

SURVEY MEASURES

Read aloud: Thank you for taking time to be part of our research study. What we discuss today is confidential.

I want to spend some time asking you some questions. The researchers have chosen to use a computer program to record your answers so that what you say cannot be accessed by anyone but the researchers.

When I record your response in the computer, it will be stored away in the computer in a secure location so that only the researchers can access it later. NO ONE ELSE will know your answers. Also, you will NOT be asked to give your name during this survey, so you can be assured that your answers will remain completely confidential.

The information in the survey is not given to anyone in your community and will be used only for research purposes. Please be honest when you answer the questions. By being honest you will help the researchers improve services in this community.

ENGAGEMENT IN HIV CARE AMONG FISHERFOLK

INFORMATION ENTERED BY INTERVIEWER

PARTICIPANT STUDY ID	☐☐☐☐☐

Interviewer: Indicate if the participant’s HIV card has the following information:

Enter clinic ID: ________________

Read aloud: Nkwebaza olw’okuwaayo obudde newetaba mu kunoonyereza kuno. Bye tugenda okwogerako leero bigenda kuba bya kyaama.

Nandyagadde okutwala akaseera nga nkubuzaayo ku bibuuzo. Abanoonyereza basazeewo okuwandiika by’onaddamu ku kompyuta okulaba nti tebituukibwako muntu mulala yenna okuleka abanoonyereza bokka.

Bwe mpandiika ebyo by’onanziramu ku kompyuta, bijja ku kwekebwa ku kompyuta mu kafo akekusifu okusobozesa abanoonyereza bokka okubikozesa oluvannyuma. TEWALI MUNTU MULALA YENNA agenda kumanya by’onanziramu. Ekirala, tetujja kukubuuza linnya lyo mu kunoonyereza kuno, okwongera okukukakasa nti by’onaddamu bijja kusigalira ddala nga bya kyaama.

Bwino gwetujja mu kunoonyereza tagenda kuweebwa muntu yenna mu kitundu kyo, era agenda kukozesebawa mu bya kunoonyereza byokka. Tukusaba obeere w’amazima ng’oddamu ebibuuzo. Mu kubeera ow’amazima kijja kuyamba abasawo abali mu kunoonyereza okutumbula empeereza z’ebyobulamu mu kitundu kino.

DEMOGRAPHICS AND SES

First I'm going to ask you some basic questions about yourself
Okusookela ddala ngenda kukubuuza ebibuzo ebyabulijjo ebikukwatako ng' omuntu

DEM1	What is your age?	
DEM1	Olina emyaka emeka?	
DEM2	What is your tribe?	Muganda (0)<br>Munyarwanda<br>Munyoro<br>Murundi<br>Mukiga<br>Mutooro<br>Munyankole<br>Musoga<br>Other (6)
DEM2	Oli wa ggwanga ki?	Muganda (0)<br>Munyarwanda<br>Munyoro<br>Murundi<br>Mukiga<br>Mutooro<br>Munyankole<br>Musoga<br>Eggwanga eddala (6)
DEM3	What is your religion?	Protestant (Anglican, Lutheran, Church of Uganda) (0)<br>Catholic<br>Moslem<br>Seventh Day Adventist<br>Saved/Pentecostal<br>None<br>Other
DEM3	Oli wa / Osoma ddiini ki?	Mukulisitayo (Anglican, Lutheran, Church of Uganda) (0)<br>Mukatoliki<br>Musiraamu<br>Musevenisidde<br>Mulokole<br>Sirina ddiini<br>Eddini endala (6)
DEM4	Are you currently married, separated, widowed, divorced, or have you never been married? (Use participant's own definition of marriage)	Married and living together most of the time. (4)<br>Married and separated most of the time (3)<br>Widowed (2)<br>Divorced (1)<br>Never married (0)
DEM4	Mu kiseera kino oli mufumbo, mwayawukana, oli ssemwandu,oba towasangako (Use participant's own definition of marriage)	Mufumbo era babeera bonna ebiseera ebisinga. (4)<br>Mufumbo era tababeera bonna ebiseera ebisinga (3)<br>Ssemwandu (2)<br>Bayawukana (1)<br>Tawasangako (0)
DEM5	How many women do you consider your wife? (If not married enter zero).	If not married response must be 0.
DEM5	Abakyaala bameka botwaala nga bakazibo? (if not married enter zero)	If not married response must be 0.
DEM6	Are you circumcised?	Yes (1)<br>No (0)
DEM6	Wakomolebwa?	Yye (1)<br>Nedda (0)

DEM7	What is your occupation?	Fisherman (0)<br>Fish seller/trader<br>Fish cleaner<br>Fish dryer<br>Boat operator (4)<br>Boat repairer/maker (5)<br>Loader (6)<br>Other occupation supporting the fish industry _________(fill in) (7)
DEM7	Okola mulimu ki?	Oli muvubi (0)<br>Otunda byennyanja/ musuubuzi wa byennyanja<br>Olongoosa byennyanja<br>Okaza byennyanja<br>Omugoba we lyaato (4)<br>Abakola oba oukanika amaato (5)<br>Abasitula ebintu (6)<br>Ekirala occupation supporting the fish industry (7) _________(fill in)
DEM8	What are your typical working hours?	Week days, daytime (0)<br>Week days, night time<br>Weekends, daytime<br>Weekends, night time<br>All week, at night<br>All week, daytime (5)
DEM8	Biseera ki mw'otera okukoleramu emirimu gyo?	Ku nnaku za wiiki, emisana (0)<br>Ku nnaku za wiiki, ekiro<br>Ku wiikendi, emisana<br>Ku wiikendi, ekiro (3)<br>Wiiki yona ekiro<br>Wiiki yona emisana (5)
DEM9	What is the highest level of school you completed?	No schooling (0)<br>P1-P7<br>S1-S4<br>S5-S6<br>Tertiary/Vocational<br>University or greater (5)
DEM9	Wakoma ku ddaala ki ery'obuyigirize?	Saasoma (0)<br>P1-P7<br>S1-S4<br>S5-S6<br>Ttendekero lya wagulu/ ery'emikono<br>Yunivasite oba okusingawo (5)
DEM10	Now I would like you to read this sentence to me. (show the literacy card to the subject) *"Clap your hands and count to ten"*	Cannot read at all (0)<br>Able to read only part of the sentence (1)<br>Able to read whole sentence (2)<br>Card was not in subject's language (3)
DEM10	Kati nandyagade onsomere ebintu bino. (show the literacy card to the subject) *"Kuba mu ngalo era obale okutuuka ku kumi"*	Cannot read at all (0)<br>Able to read only part of the sentence (1)<br>Able to read whole sentence (2)<br>Card was not in subject's language (3)
DEM11	During the last year how often did you work for money?	Throughout the year (3)<br>Part of the year (2)<br>Once in a while (1)<br>Never (0)
DEM11	Mu mwaka oguyise, emirundi emeka lwe wakolanga emirimu n'ekigendererwa ky'okufuna ensimbi?	Mu mwaka gwonna (3)<br>Ekitundu ky'omwaka (2)<br>Lumu na lumu (1)<br>Tekibangawo(0)
DEM12	How much do you usually earn per month?<br>Otera kufuna sente nga meka buli mwezi?	
DEM12	Otera kuyingiza sente nga meka buli mwezi	

Mobility

I would like to next ask you some questions about how often you shift to other communities.
Njagala Kudako okubuuza ku bikwaata ku di lwotela okusenguka mu bitundu ebilala

MOB1.	How long have you worked in this location /landing site?	Less than 6 months (0)<br>6-12 months<br>Between 1-5 years<br>5+ years<br>All my life<br>Don’t work here (5)
MOB1	Omaze banga ki ng’okolera mu kitundu kino/ ku mwalo guno?	Sisuka myezi 6 (0)<br><br>Wakati w’emyezi mukaaga ne kumi n’ebiri (6-12 months)<br><br>Wakati w’omwaka gumu n’etaano (1-5 years) (2)<br><br>Emyaka etaano n’okusingawo. (5+ years) (3)<br><br>Obulamu bwange bwonna (4)<br><br>Sikolera wano (5)
MOB2.	How long have you lived in this community?<br><br>Skip next 4 questions if mob2=4 (all my life)	Less than 6 months (0)<br>6-12 months<br>Between 1-5 years<br>5+ years<br>All my life (4)<br>Doesn’t live here (5)
MOB2	Omaze banga ki ng’obeera mu kitundu kino?	Sisuka myezi 6 (0)<br><br>Wakati w’emyezi mukaaga ne kumi n’ebiri (6-12 months)<br><br>Wakati w’omwaka gumu n’etaano (1-5 years) (2)<br><br>Emyaka etaano n’okusingawo. (5+ years) (3)<br><br>Obulamu bwange bwonna (4)<br><br>Sibeela wano (5)
MOB3.	How far from this community were you living before you came here?	Less than 10 KM (0)<br>10-29 KM<br>30-49 KM<br>50-99 KM<br>100 KM+ (4)<br>Don’t knw (5)
MOB3	Waliwo olugendo lwa buwanvu ki okuva mu kitundi kino ne gyewava?	Obutasuka kilomita 10 (0)<br>Kilomita 10 ku 29<br>Kilomita 30 ku 49<br>Kilomita 50 ku 99<br>Kilomita 100 n’okusukkawo (4)<br>Simanyi (5)

MOB4.	Can you tell me how far away they were living in terms of hours?	Enter hours _______
MOB4.	Osobola okumbulira obuwanvu bw'olugendo mu ngeri y'essaawa z'omala mu lugendo?	Essaawa __________
MOB5.	Why did you leave that place to come here?	Employment/Business (0)<br>Came back to birth place (1)<br>Got married here (2)<br>Was seeking for better food (3) supply<br>Political instability (4)<br>Came with parents (5)<br>High crime (6)<br>Fish availability (7)<br>Other _________ (8)
MOB5.	Lwaki wasalawo okuva mu kifo ekyo nojja wano?	Okukola/ Bizinensi (0)<br>Nakomawo waka gyenzalwa (1)<br>Nawasa wano (2)<br>Nali noonya wensobola okugya emmere emala (3)<br>Obutabanguko/ entalo (4)<br>Najja ne bazadde bange (5)<br>Ebikolwa byekitujju ebisusse<br>Okubaawo kwebyenyanja<br>Ekirala _________)
MOB6.	In the past 12 months, have you travelled and slept away from this community? Skip to end if no	Yes<br>No
MOB6.	Mu myezi 12 egiyise, otambudeko n'osula ebweru w'ekitundu kino?	Yye<br>Nedda
MOB7.	In the past 12 months, how many times did you travel and sleep away from this community?	Once (0)<br>2-5 Times<br>6-10 Times<br>10+ Times (3)
MOB7.	Mu myezi 12 egiyise, emirundi emeka gye watambulako n'osula ebweru w'ekitundu kino?	Omulundi gumu (0)<br>Emirundi ebiri kw'etaano<br>Emirundi mukaaga ku kumi<br>Emirundi kumi n'okusingawo (3)
MOB8.	How far from here is the most recent place you travelled and slept (enter in hours)?	Enter hours _______
MOB8.	Waliwo buwanvu ki okuva mu kitundi kino okutuuka mu kitundi gye wakasemba okugenda n'osulayo? (enter in hours)	Essaawa………………
MOB9.	What is the primary reason you leave this community?	Fishing on the lake (0)<br>Other business reasons<br>Visiting family or loved ones<br>Other _____(3)
MOB9.	Nsonga ki enkulu ekujja mu kitundu kino?	Okugenda okuvuba ku nnyanja (0)<br>Ensonga za bizinesi endala<br>Okukyalira abenganda n'abemikwano<br>Ensonga endala……… (3)
MOB10.	How long are you typically away from the community when you travel?	Less than a week (0)<br>2-3 weeks<br>A month<br>2-3 months<br>4-6 months<br>More than 6 months (5)
MOB10.	Otera kumala banga ki nga toli mu kitundu kino bw'obeera otambudemu	Obutasuka wiiki (0)<br>Wiiki bbiri ku sattu<br>Omwezi gumu<br>Emyezi ebiri ku ebiri<br>Emyezi ena ku mukaaga<br>Okusuka mu myezi mukaaga (5)

HIV Clinic Attendance and CD4 Testing

Interviewer: Ask patient to present to you their HIV clinic card. Ask them the questions and verify their answer from their HIV clinic card.

First, I would like to ask you questions about going to the HIV clinic.

Okusookera ddala, nandyagade okukubuuza ebibuuzo ebikwata ku by'okugenda ku ddwaliro erijjanjaba akawuka.

HIVCLIN1.	How long does it take you to get to the clinic? (one way)<br>*Interviewer: enter response in minutes*	Minutes_______
HIVCLIN1.	Kikutwalira banga ki okutuuka ku ddwaliro/ kiriniki? (mu lugendo olumu)	Eddakiika ………………..
HIVCLIN2.	When did you learn that you were HIV positive?	Enter Month/year ___/___
HIVCLIN2.	Wakimanya ddi nti olina akawuka?	Omwezi/omwaka ……/…….
HIVCLIN3.	What form of transportation do you usually take to get to the clinic?	Walking (1)<br>Bicycle (2)<br>Taxi(3)<br>boda boda (4)<br>Privately owned car (5)<br>Special hire (6)<br>Boat/ferry (7)
HIVCLIN3.	Ntambula ya kika ki gy'otera okukozesa okugenda ku ddwaliro/ kiriniki?	Okutambuza ebigere (1)<br>Akagaali (2)<br>Takisi (3)<br>Bodaboda (4)<br>Emmotoka y'obwananyini (5)<br>Okupangisa sipeesulo (6)<br>Lyaato oba kidyeeli (7)
HIVCLIN4.	When you tested positive for HIV, where did you get tested?	In a health facility (0)<br>In a community testing event (1)<br>In the home (door-to-door/home-based testing) (2)<br>Self-testing kit (3)
HIVCLIN4.	Bwe wakeberebwa n'osangibwamu akawuka, wa gye bakukeberera?	Mu kifo ky'obujjanjabi (0)<br>Mu lukuŋaana lw'okukebera omusaayi mu kitundu (1)<br>Ewaka (okukebera omusaayi okw'enju ku nju) (2)<br>Neekebeza nzekka ne kiiti (3)

NoOfVis6. Interviewer: Record the number of HIV clinic visits scheduled/recorded on the participants HIV card in the prior 6 months. If the clinic card is missing or incomplete, use self-report. ________

NoOfVis. Interviewer: Record the number of HIV clinic visits scheduled/recorded on the participants HIV card in the prior year. If the clinic card is missing or incomplete, use self-report. __________

If noofvis =0 , skip to question 13.

Interviewer: *For each visit reported in the last year, indicate whether if the visit was on schedule*

Visit 1: Was the visit on schedule, yes or no?
Visit 2: Was the visit on schefule, yes or no?
Visit 3: Was the visit on schefule, yes or no?
Visit 4: Was the visit on schefule, yes or no?
Visit 5: Was the visit on schefule, yes or no?

Visit 6: Was the visit on schefule, yes or no?<br>Visit 7: Was the visit on schefule, yes or no?<br>Visit 8: Was the visit on schefule, yes or no?<br>Visit 9: Was the visit on schefule, yes or no?<br>Visit 10: Was the visit on schefule, yes or no?

10. What was the main reason(s) you missed or came late to your most recent missed visit?

Nsonga ki eyakuleetela okuba nga tewagya oba wagya kikeelezi kulunaku lwebaakuwa okugyilako?

Put N/A if all visits were kept on schedule

Lack of transport (0)

Forgot

Was far away from home

Afraid of stigma

Afraid the providers will tell people you have HIV

Illness/Hospitalization

Afraid to be seen at the HIV clinic

Don't like the providers at the HIV clinic

Had a bad experience at previous clinic visit

Did not feel sick

Was fishing on the lake

Work hours conflicted with clinic times (11)

Others (12) (HIVCLN10o. Specify)_________________________

N/A (no visits missed)

10. If yes, nsonga ki (eza)eyakuviirako obutagenda ku ddwaliro ku mulundi ogwakasembayo?

Okubulwa entambula (0)

Okwerabira (1)

Wali wala nnyo okuva n'ewaka (2)

Okutya okusongebwamu olunnwe (3)

Okutya nti abasawo bajja kugamba abantu nti olina akawuka (4)

Wali mulwadde/ wali ku kitanda (5)

Okutya okulabibwa ku ddwaliro/ kirinika y'abalina akawuka (6)

Abasawo abali ku ddwaliro/ kirinika y'abalina akawuka tobaagala (7)

Wayita mu mbeera embi ku mulundi gwewasemba okugenda ku ddwaliro/ kirinika y'abalina akawuka (8)

Wali owulira nga toli mulwadde (9)

Wali ovuba ku nnyanja (10)

Obudde bw'okukola bwali tebukwatagana na budde bwa kugenda ku ddwaliro/ kiriniki (11)

Ekirala (12) (12o. specify)……………………………………….

HIVCL…

	Interviewer: is there a CD4 test result in the participants file?	No, and participant said they never had one taken (Skip CD4 questions)<br>No, but participant said they have had one taken<br>Yes
13.	Interviewer: record the dates of the last CD4 counts	Enter Month/year ___/___

14.	Interviewer: Record the CD4 results	
15.	Interviewer: Is there a viral load result in the file?<br><br>*If HIVCLIN15 = 0*<br>*Interviewer: If participant has never had a viral load taken, be sure to refer them to clinic staff to have viral load taken today*	No, and participant said they never had one taken (Skip vl questions)<br>No, but participant said they have had one taken<br>Yes
16.	Interviewer: Enter the date of the most recent viral load test	Enter Month/year ___/___
17.	Interviewer: Enter the viral load result	Enter VL ______

Adherence measures

I am going to ask you some questions about the ARVs that you are currently taking.

Kati ŋenda ku kubuuza ebibuuzo ebikwata ku ddagala lya ARV ly'omira mu kiseera kino.

ARV1	Interviewer: Record when the client was first prescribed ARVs ever.<br><br>*Confirm date with patient.*	______ / ______<br>Month Year

ARVReg	Interviewer: What regimen is the participant on?	(0) First line therapy<br>(1) Second line therapy<br>(2) Other
ARV2a	**Ask next 3 questions only for 1st line regimen patients**<br>Interviewer: which first line regimen is the participant on?	(0) Preferred regimen, TDF+3TC+EFV<br>(1) Alternate regimen: AZT +3TC +EFV<br>(2) Alternate regimen: AZT +3TC +NVP<br>(3) Alternate regimen: TDF +3TC +NVP
ARV2c	How many times per day do you take your ARVs?<br><br>Emilundi emeka gyomila empeke zo eza ARV buli lunaku?	Enter number
ARV2b	Each time you take you ARVs, how many pills do you take at one time?<br><br>Buli lwomila empeke zo eza ARVs, omila empeke meka omulindu gumu	Enter number
ARV3a	**Ask next 3 questions only for 2nd line regimen patients**<br>Interviewer: Which second line regimen is the participant on?	(0) TDF/3TC<br>(1) AZT+3TC<br>(2) TDF+3TC<br>(3) Backbone NRTI<br>(4) Heat stable ATV/r the preferred PI of choice
ARV3b	How many times per day do you take your ARVs?<br><br>Emilundi emeka gyomila empeke zo eza ARV buli lunaku_________?	Enter number

ARV3c	Each time you take you ARVs, how many pills do you take at one time _________?<br><br>Buli lwomila empeke zo eza ARVs, omila empeke meka omulindu gumu_________?	Enter number
ARV4a	**Ask next 3 questions only for 3rd line regimen patients or "other" regimen**<br><br>Interviewer: What "other" regimen is the participant on?	(0) Third line: Darunavir/r + Raltegravir (Etravirine) + TDF and 3TC<br>(1) Other (fill in:____)
ARV4c	How many times per day do you take your ARVs?<br><br>Emilundi emeka gyomila empeke zo eza ARV buli lunaku_________?	Enter number
ARV4b	Each time you take you ARVs, how many pills do you take at one time _________?<br><br>Buli lwomila empeke zo eza ARVs, omila empeke meka omulindu gumu_________?	

We understand that many people on ARVs find it difficult to take these medications every day, and there are times when people miss their doses or forget to take their medications. We would not be surprised if you have sometimes missed your doses or forgotten to take your ARVs as well.

I would like to know how much of each of your ARVs you <u>missed</u> yesterday, two days ago, three days ago, and four days ago. If you took only a <u>portion</u> of a dose on one or more of these days, please report the doses(s) as being <u>missed</u>.

Tukitegeera nti abantu bangi abali ku ddagala lya ARV basanga obuzibu mu kumira eddagala buli lunaku, era waliwo ebiseera lwe bayosa okumira ddoozi zaabwe oba lwebeerabira okumira eddagala lyabwe. Tetuyinza kwewunya bwoba ne ddoozi zewabuukamu oba nga werabira okumira eddagala lyo erya ARV emirundi egimu.

Nandyagadde okumanya ku bungi bw'eddagala lyo erya ARV lyotaamira olunaku lwejjo, enaku biri emabega, enaku satu emabega n'enaku nnya emabega. Nebwoba wamirako kitundu ku ddoozi yo ku zimu ku naku zino, nkusaba eyo/ ezo ddoozi ozibale nga eyazibuuka

First line therapy

ARV2d	How many of dose of your ARVs did you miss <u>yesterday</u>?	
ARV2d	Wabuusemu ddoozi meka eza ARV olunaku lw'ajjo?	
ARV2e	How many of these doses did you miss <u>two days ago</u>?	
ARV2e	Ku ddagala lino, ddoozi meka zewabuukamu (ku lunaku oluli) enaku biri emabega?	
ARV2f	How many of these doses did you miss <u>3 days ago</u>?	
ARV2f	Ku ddagala lino, ddoozi meka zewabuukamu (ku lunaku oluli) enaku satu emabega?	
ARV2g	How many of these doses did you miss <u>4 days ago</u>?	
ARV2g	Ku ddagala lino, ddoozi meka zewabuukamu (ku lunaku oluli) enaku nnya emabega?	
ARV17	During the past 4 days, on how many days have you missed taking <u>all of your doses?</u>	None (0)<br>One day<br>Two days<br>Three days

		Four days (4)
ARV17	Mu naku nnya eziyise, naku meka kw'otamiride yadde ddoozi n'emu?	Tewali (0)<br>Olunaku lumu<br>Enaku biri<br>Enaku satu<br>Enaku nnya (4)
ARV18	In the last four days, how often did take the right number of pills at the right time, as prescribed?	Never<br>Some of the time<br>About half of the time<br>Most of the time<br>All of the time
ARV18	Mu nakku nya eziyise, obadde otela okumila eddagala lyo mubutuufu bwaalyo ne mu budde obuutufu nga abasawo bwe bakulagidde?	Tekibeerangawo yadde (0)<br>Ebiseera ebimu<br>Kumpi kitundu ky'ebiseera byonna<br>Ebiseera ebisinga<br>Ebanga lyonna (4)
ARV19	Do any of your ARV medications have special instructions, such as "take with food" or "on an empty stomach" or "with plenty of fluids?"	Yes<br>No → **skip next question**
ARV19	Ku ddagala lyo erya ARV kuliko eririna ebiragiro eby'enjawulo, okugeza "ery'okumirirako emmere" oba "ery'okumira nga tonnalya kintu kyonna" oba "erikwetaaza okunywa ennyo"?	Yye<br>Nedda→ **skip next question**
ARV19b	If yes, how often did you follow these special instructions over the last four days?	Never<br>Some of the time<br>About half of the time<br>Most of the time<br>All of the time
ARV19b	Otela kusa ebiragiro bino mu nkola mu naku nnya eziyise?	Tekibeerangawo yadde (0)<br>Ebiseera ebimu<br>Kumpi kitundu ky'ebiseera byonna<br>Ebiseera ebisinga<br>Ebanga lyonna (4)
ARV20	Did you miss any of your ARV medications last weekend— last Saturday or Sunday? Some people find that they forget to take their pills on the weekend days.	Yes<br>No
ARV20	Ku wiikendi ewedde (ku lwomukaaga oluwedde oba ku sande ewedde) waliwo eddgala lyo erya ARV lyonna lyotaamira? Abantu abamu besanga nga berabidde okumira eddagala lyabwe ku naku za wiikendi.	Yye<br>Nedda
ARV21	When was the last time you missed any of your medications?	In the past week (0)<br>In the past 4 weeks<br>Between 1 and 3 months ago<br>More than 3 months ago<br>You have never missed taking any of your ARVs (4)→ **skip next section**
ARV21	Wasemba ddi okwosa mu kumira eddagala lyo ery'ekika kyonna?	Mu wiiki emu eyise (0)<br>Mu wiiki nnya eziyise<br>Wakati wa wiiki emu n'emyezi esatu emabega<br>Okusuka mu myezi esatu<br>Toyosanga mu kumira eddagala lyo erya ARV lyonna (4)→ **skip next section**
ARV22	[IF ON 2ND OR 3RD LINE THERAPY]<br>Why was your regimen changed (from 1st to 2nd line, or from 2nd to 3rd line therapy)	(0) Treatment failure (i.e., problems with adherence)<br>(1) Problems with side effects/tolerance<br>(2) I don't know (ARV22dk: _____ fill in from HIV card)

ARV22	[Bwaba ali ku layini y'obujanjabi eyokubiri]<br>Lwaki bakukyusa okuva ku layini y'obujanjabi esooka okukuteeka ku layini y' obujanjabi ey' okubiri	(0) ebuzibu n'okunywerera ku mira y' eddagala<br>(1) obuzibu nebiva mu nkyukakyuka z' okumira eddagala oba okugumira ebiva mu kumira eddagala<br>(2) Simanyi

People may miss taking their medications for various reasons. Here is a list of possible reasons why you may miss taking your medications. How often have you missed taking your medications because you:

Abantu basobola okwosa mu kumira eddagala lyabwe olw'ensonga ez'enjawulo. Luno olukalala lulaga ensonga ezisobola okukuviirako okwosa mu kumira eddagala lyo. Emirundi emeka lwoyoseza mu kumira eddagala lyo olwokuba:

ADH1	Were away from home?	Never (0)<br>Rarely (1)<br>Sometimes (2)<br>Often (3)
ADH1	Wali wala okuva ewaka?	Tekibeerangawo (0)<br>Tekitera kubeerawo (1)<br>Olumu kibeerawo (2)<br>Kibeerawo emirundi egiwera (3)
ADH2	Were busy fishing on the lake?	Never<br>Rarely<br>Sometimes<br>Often
ADH2	Wali oli mu kuvuba ku nnyanja?	Tekibeerangawo<br>Tekitera kubeerawo<br>Olumu kibeerawo<br>Kibeerawo emirundi egiwera
ADH3	Were busy with other things?	Never<br>Rarely<br>Sometimes<br>Often
ADH3	Walina ebintu ebirala byewali okola ebyakufuula bizze?	Tekibeerangawo<br>Tekitera kubeerawo<br>Olumu kibeerawo<br>Kibeerawo emirundi egiwera
ADH4	Simply forgot?	Never<br>Rarely<br>Sometimes<br>Often
ADH4	Werabira bwerabizi?	Tekibeerangawo<br>Tekitera kubeerawo<br>Olumu kibeerawo<br>Kibeerawo emirundi egiwera
ADH5	Had too many pills to take?	Never<br>Rarely<br>Sometimes<br>Often
ADH5	Walina empeke nyinji ez'okumira?	Tekibeerangawo<br>Tekitera kubeerawo<br>Olumu kibeerawo<br>Kibeerawo emirundi egiwera
ADH6	Wanted to avoid side effects?	Never<br>Rarely<br>Sometimes<br>Often
ADH6	Wali ogezaako kwewala zi sayidifekiti/ obulabe obuva mu kumira eddagala?	Tekibeerangawo<br>Tekitera kubeerawo<br>Olumu kibeerawo

		Kibeerawo emirundi egiwera
ADH7	Did not want others to notice you taking medication?	Never<br>Rarely<br>Sometimes<br>Often
ADH7	Wali toyagala bantu balala kukitegeera nti omira eddagala?	Tekibeerangawo<br>Tekitera kubeerawo<br>Olumu kibeerawo<br>Kibeerawo emirundi egiwera
ADH8	Had a change in daily routine?	Never<br>Rarely<br>Sometimes<br>Often
ADH8	Wafuna onkyukakyuka mu ngeri gy'okolamu ebintu byo ebyabulijjo?	Tekibeerangawo<br>Tekitera kubeerawo<br>Olumu kibeerawo<br>Kibeerawo emirundi egiwera
ADH9	Felt like the drug was toxic/harmful?	Never<br>Rarely<br>Sometimes<br>Often
ADH9	Eddagala waliwuliranga ery'obutwa/ ery'obulabe?	Tekibeerangawo<br>Tekitera kubeerawo<br>Olumu kibeerawo<br>Kibeerawo emirundi egiwera
ADH10	Fell asleep/slept through dose time?	Never<br>Rarely<br>Sometimes<br>Often
ADH10	Otulo twakubba/ webakira mu budde bw'okumiriramu eddagala?	Tekibeerangawo<br>Tekitera kubeerawo<br>Olumu kibeerawo<br>Kibeerawo emirundi egiwera
ADH11	Felt sick or ill?	Never<br>Rarely<br>Sometimes<br>Often
ADH11	Wali wewulira ng'omulwadde?	Tekibeerangawo<br>Tekitera kubeerawo<br>Olumu kibeerawo<br>Kibeerawo emirundi egiwera
ADH12	Felt depressed/overwhelmed?	Never<br>Rarely<br>Sometimes<br>Often
ADH12	Wali owulira nga wenyamidde oba ng'owulira ozitoowereddwa	Tekibeerangawo<br>Tekitera kubeerawo<br>Olumu kibeerawo<br>Kibeerawo emirundi egiwera
ADH13	Had problems taking pills at specified times (with meals, on an empty stomach, etc.)?	Never<br>Rarely<br>Sometimes<br>Often
ADH13	Wasanga obuzibu mu kumira eddagala lyo mu biseera ebukuweereddwa (okulimiririrako emmere, okulimira ng'olubuto lukalu, nebirala)?	Tekibeerangawo<br>Tekitera kubeerawo<br>Olumu kibeerawo<br>Kibeerawo emirundi egiwera
ADH14	Ran out of pills	Never<br>Rarely<br>Sometimes<br>Often
ADH14	Empeke zali zikuweddeko	Tekibeerangawo<br>Tekitera kubeerawo<br>Olumu kibeerawo<br>Kibeerawo emirundi egiwera
ADH15	Felt good/healthy?	Never

		Rarely<br>Sometimes<br>Often
ADH15	Wali wewulira bulungi/ ng'obulamu tebulina buzibu/ tolina wakuluma?	Tekibeerangawo<br>Tekitera kubeerawo<br>Olumu kibeerawo<br>Kibeerawo emirundi egiwera
ADH16	Forgot because of alcohol or drug use?	Never<br>Rarely<br>Sometimes<br>Often
ADH16	Werabira olwokuba wali onyweddemu ku mwenge oba olw'okukozesa ebiragalalagala?	Tekibeerangawo<br>Tekitera kubeerawo<br>Olumu kibeerawo<br>Kibeerawo emirundi egiwera
ADH17	Did not want to mix alcohol or drugs with pills?	Never<br>Rarely<br>Sometimes<br>Often
ADH17	Wali toyagala kugatika mwenge oba ebiragalalagala na ddagala?	Tekibeerangawo<br>Tekitera kubeerawo<br>Olumu kibeerawo<br>Kibeerawo emirundi egiwera
ADH18	Could not get pills because work hours conflict with clinic hours?	Never<br>Rarely<br>Sometimes<br>Often
ADH18	Tewasobola kufuna ddagala kubanga obudde bw'okugenda ku ddwaliro/ kiriniki buyingira mu budde bw'okukola?	Tekibeerangawo<br>Tekitera kubeerawo<br>Olumu kibeerawo<br>Kibeerawo emirundi egiwera
ADH19	Ran out of drugs because did not want to be seen at the clinic?	Never<br>Rarely<br>Sometimes<br>Often
ADH19	Eddagala lyakuggwako kubanga wali toyagala kulabibwa webajanjabila?	Tekibeerangawo<br>Tekitera kubeerawo<br>Olumu kibeerawo<br>Kibeerawo emirundi egiwera
ADH20	Wanted to avoid interactions/side effects with other medicine you are taking?	Never<br>Rarely<br>Sometimes<br>Often
ADH20	Wali toyagala kufuna kugatilila oba kufuna nkyukakyuuka ne ddagala eddala lyomira?	Tekibeerangawo<br>Tekitera kubeerawo<br>Olumu kibeerawo<br>Kibeerawo emirundi egiwera

ALCOHOL USE DISORDERS (AUDIT)

Now I have some questions about alcohol. Alcohol includes **beer, marwa, waragi, tonto**, **wine** or **any beverage that contains alcohol.** Please **do not include** communion wine or wine that you received at church when answering these questions.

Kati nanawo ebibuuzo ebitontono ebikwata ku mwenge. Mu mwenge, tulinamu **bbiya**, **amalwa**, **walagi**, **ttonto**, **wayini** oba **ekyokunnywa kyonna kyebatabiseemu omwenge**.

ALC1	How often do you have a drink containing alcohol? Never, less than monthly, monthly, weekly, or daily or mostly daily?	Never (0)<br>Less than once a month<br>At least once a month<br>At least once a week<br>Every day (4)
ALC1	Emirundi emeka lw'otera okunnywa ekyokunnywa ekirimu omwenge? Tekibangawo, obutassuka mulundi gumu mu mwezi, omulundi gumu mu wiiki, buli lunaku, okumala olunaku lwonna.	Tekibangawo (0)<br>Obutasukka mulundi gumu mu mwezi<br>Wakiri omulundi gumu mu mwezi<br>Wakiri omulundi gumu mu wiiki<br>Buli lunaku (4)
ALC2	(Besides wine such as you may have received at church), have you ever drunk alcohol?	Yes<br>No
ALC2	(Ng'ogyeko wayini ng'oyo anywebwa mu kkanisa), wali onywedde ku mwenge?	Yye<br>Nedda
ALC3	Have you drunk alcohol during the **last 12-months**?	Yes<br>No (skip remaining questions in this section)
ALC3	Onyweddeko ku mwenge mu myezi 12 egiyise?	Yye<br>Nedda (skip remaining questions in this section)
ALC4	How many drinks of any kind containing alcohol do you have on a typical day when you are drinking? (show participant drink sizes)	1 to 2 (0)<br>3 to 4<br>5 to 6<br>7, 8, or 9<br>10 or more (4)
ALC4	Mu lunaku onywa ebyokunywa ebirimu omwenge bimeka? Ebyokunywa bino bisobola okuba eby'ekika kyonna. (show participant drink sizes)	1 ku 2 (0)<br>3 ku 4<br>5 ku 6<br>7, 8, oba 9<br>10 oba n'okusingawo (4)
ALC5	How often do you have six or more drinks of any kind on one occasion? Never, less than monthly, monthly, weekly, or daily or mostly daily?	Never (0)<br>Less than once a month<br>At least once a month<br>At least once a week<br>Every day (4)
ALC5	Emirundi emeka lw'otera okunywa ebyokunywa mukaaga oba okusukkawo mu lunnywa lumu? Ebyokunywa bino bisobola okuba eby'ekika kyonna.	Tekibangawo (0)<br>Obutasukka mulundi gumu mu mwezi<br>Wakiri omulundi gumu mu mwezi<br>Wakiri omulundi gumu mu wiiki<br>Buli lunaku (4)
ALC6	How often during the last **year** have you found that you were not able to stop drinking once you had started?	Never (0)<br>Less than once a month<br>At least once a month<br>At least once a week<br>Every day (4)
ALC6	Mu mwaka oguyise, kikubaddeko emirundi emeka lwewesanze nga tosobola kulekeraawo kunnywa oluvannyuma lw'okutandika okugunywa?	Tekibangawo (0)<br>Obutasukka mulundi gumu mu mwezi<br>Wakiri omulundi gumu mu mwezi<br>Wakiri omulundi gumu mu wiiki<br>Buli lunaku (4)
ALC7	How often during the last **year** have you failed to do what was normally expected from you because of drinking?	Never (0)<br>Less than once a month<br>At least once a month

		At least once a week<br>Every day (4)
ALC7	Mu mwaka oguyise, emirundi emeka lw'olemeddwa okukola ebyo ebikusuubirwamu olw'ettamiiro?	Tekibangawo (0)<br>Obutasukka mulundi gumu mu mwezi<br>Wakiri omulundi gumu mu mwezi<br>Wakiri omulundi gumu mu wiiki<br>Buli lunaku (4)
ALC8	How often during the last **year** have you needed a drink first thing in the morning to get yourself going after a heavy drinking session?	Never (0)<br>Less than once a month<br>At least once a month<br>At least once a week<br>Every day (4)
ALC8	Mu mwaka oguyise, kikubaddeko emirundi emeka lwe wetaaze okusooka okunywamu ng'ekintu ekisookera ddala kumakya osobole okutambuza obulamu oluvannyuma lw'okunnywa omwenge omungi?	Tekibangawo (0)<br>Obutasukka mulundi gumu mu mwezi<br>Wakiri omulundi gumu mu mwezi<br>Wakiri omulundi gumu mu wiiki<br>Buli lunaku (4)
ALC9	How often during the last **year** have you had a feeling of guilt or remorse after drinking?	Never (0)<br>Less than once a month<br>At least once a month<br>At least once a week<br>Every day (4)
ALC9	Mu mwaka oguyise, kikubaddeko emirundi emeka lwe weewuliddemu okwesalira omusango oba okwejjusa oluvannyuma lw' okunnywa omwenge?	Tekibangawo (0)<br>Obutasukka mulundi gumu mu mwezi<br>Wakiri omulundi gumu mu mwezi<br>Wakiri omulundi gumu mu wiiki<br>Buli lunaku (4)
ALC10	How often during the last **year** have you been unable to remember what happened the night before because you had been drinking?	Never (0)<br>Less than once a month<br>At least once a month<br>At least once a week<br>Every day (4)
ALC10	Mu **mwaka** oguyise, emirundi emeka lw'olemeddwa okujjukira ebyo ebyabaddewo ekiro ekiyise olwokuba wali onywa?	Tekibangawo (0)<br>Obutasukka mulundi gumu mu mwezi<br>Wakiri omulundi gumu mu mwezi<br>Wakiri omulundi gumu mu wiiki<br>Buli lunaku (4)
ALC11	How often in the past **year** did you have sex without a condom because of your drinking? Was it never, once, a few times or many times?	Never (0)<br>Once<br>A few times<br>Many times (3)
ALC11	Mu **mwaka** oguyise, emirundi emeka gyewanyumya akaboozi nga temukozeseza kapiira olw'okuba wali onywedde omwenge? Tekibeerangawo, Kyaaliwo omulundi gumu, Kyaaliwo emirundi mitono oba Kyaaliwo emirundi mingi?	Tekibangawo (0)<br>Omulundi gumu<br>Emirundi mitono<br>Emirundi mingi (3)
ALC12	Have you or someone else **ever** been injured as a result of your drinking?	Yes, during the last year<br>Yes, but not during the last<br>No, never (0)
ALC12	Wali ofunye ku bisago oba waliwo omuntu omulala yenna eyali afunye ku bisago nga kivudde mu kunnywa kwo?	Yye, mu mwaka oguyise (2)<br>Yye, naye si mu mwaka oguyise<br>Nedda, tekibangawo (0)
ALC13	Has a relative or friend or a doctor or another health worker **ever** been concerned about your drinking or suggested you cut down?	Yes, during the last year (2)<br>Yes, but not during the last<br>No, never (0)
ALC13	Waliwo ow'oluganda lwo, mukwano gwo, oba omusawo oba omukozi w'ebyobulamu omulala yenna eyali afuddeyo/ neyeraliikirira olw'omwenge gw'onywa, oba n'akuwa amagezi okugukendeeza?	Yye, mu mwaka oguyise (2)<br>Yye, naye si mu mwaka oguyise<br>Nedda, tekibangawo (0)

Now I'm going to ask you about your drinking during the prior 30 days

Kati ŋenda kukubuuza ku by'okunywa omwenge mu naku 30 agayise.

ALC14	What do you normally take when you are drinking?	Tonto/ (0)<br>Bottled beer<br>Malwa

		Waragi<br>Any other kind of local brew<br>Any other kind of alcohol (5)
ALC14	Otera kunywa mwenge ki lw'oba onyedde?	Ttonto (0)<br>Bbiya ow'omu ccupa<br>Malwa<br>Walagi<br>Ekika ky'omwenge gw'ekinnansi ekirala<br>Ekika ky'omwenge ekirala (5)
ALC15	During the prior 30-days, on days when you drank any kind of alcohol **besides Malwa**, how many alcoholic **drinks** did you usually have in one day?<br>(1 drink = 1 bottle of beer, 1 cup of tonto a, 1 shot of waragi)	
ALC15	Mu **naku 30** egayise, ku naku kwewanyweranga ekika ky'omwenge kyonna ng'ogyeko **amalwa**, mu lunaku olumu wateranga okunnywa eby'okunnywa ebitamiiza bimeka?<br><br>(Ekyokunywa kimu = eccupa emu eya bbiya, ekikopo kimu ekya ttonto, akaveera/akagiraasi kamu aka waragi)	
ALC16	During the prior 30-days, on the days you drank Malwa, how much time did you usually spend drinking Malwa?	Hours(Saawa)
ALC16	Mu nnaku 30 egayise, wamalanga essaawa meka ng'onnywa amalwa ku nnaku ze wagannywerangako?	Essaawa……………………..

Substance Involvement: Quick Screen

If it's okay with you, I'd like to ask you a few questions that will help me give you better medical care. The questions relate to your experience with drugs. Some of the substances we'll talk about are prescribed by a doctor (like pain medications). But I will only record those if you have taken them for reasons or in doses other than prescribed. I'll also ask you about illicit or illegal drug use.

In the past year, how often have you used the following?

Bwoba okirizza, nandyagadde okukubuuza ebibuuzo ebitonotono ebijja okunnyamba okukuwa obujjanjabi obusingako obulungi. Ebibuuzo bino byekuusa ku mbeera gy'oyiseemu n'ebiragalalagala. Ebiragalalagala ebimu byetugenda okwogerako biri mu ngeri ya ddagala ng'era liwandiikiddwa omusawo (okugeza ng'eddagala eriweweeza ku bulumi). Wabula, ebiragalalagala ebigwa mu kika ekyo nja kubiwandiika bwoba obikozesezaako olw'ensonga endala eyawukana ku musawo gyeyakuwandiikira oba ng'obikozesezaako mu bungi/ emirundi egyawukana n'egy'omusawo gyeyakuwandiikira. Era nja kukubuuza ku by'okukozesa ebiragalalagala mu ngeri emenya amateeka.

Mu mwaka oguyise, ebintu bino wammanga obikozeseza emirundi emeka?

SIQS1.	Prescription drugs for non-medical reasons	Never<br>Once or twice<br>Monthly<br>Weekly<br>Daily or almost daily
SIQS1.	Eddagala erikuwandiikiddwa abasawo n'olikozesa ebintu	Tekibangawo<br>Omulundi gumu oba ebiri

	ebirala	Buli mwezi<br>Buli wiiki<br>Buli lunaku oba kumpi buli lunaku
SIQS2.	Illegal drugs	Never<br>Once or twice<br>Monthly<br>Weekly<br>Daily or almost daily<br>***If "never" to both questions, skip to next section. If ever used any drugs, continue to lifetime drug use.***
SIQS2.	Ebiragalalagala ebitakirizibwa mu mateeka	Tekibangawo<br>Omulundi gumu oba ebiri<br>Buli mwezi<br>Buli wiiki<br>Buli lunaku oba kumpi buli lunaku<br>***If "never" to both questions, skip to next section. If ever used any drugs, continue to lifetime drug use.***

Lifetime Substance Involvement

1. ***In your LIFETIME, which of the following substances have you ever used?***

1. Mu bulamu bwo bwonna, bintu ki ku bino wammanga byewali okozesezzaako?

1a.	Cannabis (marijuana, pot, grass, hash, etc.)	Yes<br>No
1a.	Cannabis (marijuana, pot, grass, hash, Njaga, irtime, ssada, kibaba, Kamwokya, bobi wine, ailey, enwa, ekika, engwi, omuti, Empuuza, bukoko, embaanga, etc.)	Yye<br>Nedda
1b.	Cocaine (coke, crack, etc.)	Yes<br>No
1b.	Cocaine (coke, crack, TABU, clear, etc.)	Yye<br>Nedda
1c.	Prescription stimulants (Ritalin, Concerta, Dexedrine, Adderall, diet pills, etc.)	Yes<br>No
1c.	Prescription stimulants (Ritalin, Concerta, Dexedrine, Adderall, diet pills, etc.)	Yye<br>Nedda
1d.	Methamphetamine (speed, crystal meth, ice, etc.)	Yes<br>No
1d.	Methamphetamine (speed, crystal meth, ice, etc.)	Yye<br>Nedda
1e.	Inhalants (nitrous oxide, glue, gas, aviation fuel, petrol, paint thinner, etc.)	Yes<br>No
1e.	Inhalants (nitrous oxide, glue, gas, amafuta g' ennyonyi, petrol, paint thinner, Leeta mubanga, gafuga obwongo,	Yye<br>Nedda

	tawootu, kawunyemu, nguumi etc.)	
1f.	Sedatives or sleeping pills (Valium, Serepax, Ativan, Xanax, Librium, Rohypnol, GHB, pilton etc.)	Yes<br>No
1f.	Sedatives or sleeping pills (Valium, Serepax, Ativan, Xanax, Librium, Rohypnol, GHB, pilton [Pilu, nsaya] etc.)	Yye<br>Nedda
1g.	Hallucinogens (LSD, acid, mushrooms, PCP, Special K, ecstasy, etc.)	Yes<br>No
1g.	Hallucinogens (LSD, acid, mushrooms, PCP, Special K, ecstasy, etc.)	Yye<br>Nedda
1h.	Street opioids (heroin, opium, etc.)	Yes<br>No
1h.	Street opioids (heroin [brown sugar, Muggo, fimbo, Akachwiri], opium, etc.)	Yye<br>Nedda
1i.	Prescription opioids (fentanyl, oxycodone [OxyContin, Percocet], hydrocodone [Vicodin], methadone, buprenorphine, morphine, etc.)<br><br>• *Please record nonmedical use only: Non-medical use refers to using a substance either not prescribed to the patient or used in ways or amounts not prescribed by their doctor.*	Yes<br>No
1i.	Prescription opioids (fentanyl, oxycodone [OxyContin, Percocet], hydrocodone [Vicodin], methadone, buprenorphine, morphine, etc.)<br><br>• *Please record nonmedical use only: Non-medical use refers to using a substance either not prescribed to the patient or used in ways or amounts not prescribed by their doctor.*	Yye<br>Nedda
1j.	Tobacco (cigarettes) or smoke pipe	Yye<br>Nedda
1j	Tobacco (cigarettes) (Sigara, Fege, kikoomi, taaba, kiika, enkongole, enyonza, ebanga, lubugo) or Smoke pipe (Mindi, kika, mitwe ena)	Yye<br>Nedda
1k.	Kuber (Kanago, kajaani, ensaano, kakuuta, mijaji)	Yye<br>Nedda
1k	Kuber (Kanago,kajaani,ensaano,kakuuta,mijaji)	Yye<br>Nedda
1l	Sisha (Kikkaka, kuba nkusekule, today tonight, embookya, akachupa, Lupiira)	Yye<br>Nedda
1l	Sisha (Kikkaka, kuba nkusekule, today tonight, embookya, akachupa, Lupiira)	Yye<br>Nedda
1m	Khati /mairungi (Kakoola, kaba, ggati, enkwakyo, kasenge, muleega waya, greens, kaba, nansivvo, kasuuwa)	Yye<br>Nedda

1m	Khati /mairungi (Kakoola, kaba,ggati,enkwakyo,kasenge,muleega waya,greens,kaba,nansivvo,kasuuwa)	Yye<br>Nedda
1n.	Other	Yes<br>No<br>(1na) If yes, specify:__________
1n.	Ekirala	Yye<br>Nedda<br>(1na) If yes, specify:__________

Recent Substance Involvement

Next, ask questions 2 through 8 for each substance the patient reported using in Question 1.

Name of substance use:

SI2 – *cannabis*

SI3 – Cocaine

SI4 - Prescription stimulants (Ritalin, Concerta, Dexedrine, Adderall, diet pills, etc.)

SI5 - Methamphetamine (speed, crystal meth, ice, etc.)

SI6 - Inhalants (nitrous oxide, glue, gas, paint thinner, etc.)

SI7 - Sedatives or sleeping pills (Valium, Serepax, Ativan, Xanax, Librium, Rohypnol, GHB, etc.)

S18 - Hallucinogens (LSD, acid, mushrooms, PCP, Special K, ecstasy, etc.)

SI9 - Street opioids (heroin, opium, etc.)

SI10 - prescription opioids (fentanyl, oxycodone [OxyContin, Percocet], hydrocodone [Vicodin], methadone, buprenorphine, etc.)

SI11 - *Tobacco (cigarettes) (Sigara, Fege, kikoomi, taaba, kiika, enkongole, enyonza, ebanga, lubugo) or Smoke pipe (Mindi, kika, mitwe ena)*

SI12 - Kuber (Kanago, kajaani, ensaano, kakuuta, mijaji)

SI13 - Sisha (Kikkaka,kuba nkusekule.today tonight,embookya,akachupa,Lupiira)

S14 - Khati /mairungi (Kakoola, kaba,ggati,enkwakyo,kasenge,muleega waya,greens,kaba,nansivvo,kasuuwa)

S15 – Ekirala

Ask the following questions for each drug mentioned in Question 1:

a.	In the past 3 months, how often have you used (insert name of drug)?	Never<br>Once or twice<br>Monthly<br>Weekly<br>Daily or almost daily<br>**If “never”, skip to Question 6. Otherwise, continue with Questions 3**

a.	Mu myezi 3 egiyise, okozeseza emirundi emeka (insert name of drug)	Tekibangawo<br>Omulundi gumu oba ebiri<br>Buli mwezi<br>Buli wiiki<br>Buli lunaku oba kumpi buli lunaku<br>**If "never", skip to Question 6. Otherwise, continue with Questions 3**
b.	In the past 3 months, how often have you had a strong desire or urge to use (insert name of drug)?	
b.	Mu myezi 3 egiyise, emirundi emeka lw'obadde n'obwagazi obw'amaanyi okukozesa (insert name of drug)	
c.	In the past 3 months, how often has your use of (insert name of drug) led to health, social, legal or financial problems?	
c.	Mu myezi 3 egiyise, emirundi emeka eky'okukozesa (insert name of drug) gyekikuviiriddeko obuzibu mu by'obulamu, obuzibu mu kutabagana n'abantu, okuvunaanibwa mu mateeka oba obuzibu mu by'ensimbi?	
d.	In the past 3 months, how often have you failed to do what was normally expected of you because of your use of (insert name of drug)?	
d.	Mu myezi 3 egiyise, emirundi emeka lw'olemeddwa okukola ekikusuubirwamu olw'okuba okozesa (insert name of drug)	

Ask Questions 12 & 13 for all substances ever used (i.e., those mentioned in Question 1):

12.	Has a friend or relative or anyone else ever expressed concern about your use of (insert name of drug)?	No<br>Yes, but not in the last 3 months<br>Yes, in the past 3 months
12.	Waliwo mukwano gwo yenna oba ow'oluganda lwo yenna eyalaga okufaayo oba eyakweralikirirako olw'okuba okozesa (insert name of drug)?	Nedda<br>Yye, naye ssi mu myezi 3 egiyise<br>Yye, mu myezi 3 egiyise
13.	Have you ever tried and failed to control, cut down, or stop using (insert name of drug)?	No<br>Yes, but not in the last 3 months<br>Yes, in the past 3 months
13.	Wali ogezezzaako okufuga, okukendeeza oba okukomya okukozesa naye n'olemwa (insert name of drug)?	Nedda<br>Yye, naye ssi mu myezi 3 egiyise<br>Yye, mu myezi 3 egiyise

Ask Question 14 if patient mentions ANY drug that might be injected, including those that might be listed in the 'Other' category (e.g., steroids).

14.	Have you ever used any drug (including steroids) by injection?<br><br>• *Indicate you are referring to non-medical use only.*	No<br>Yes, but not in the last 3 months<br>Yes, in the past 3 months

14.	Wali okozesezzaako ku kika ky'ebiragalalagala kyonna (ng'otaddemu n'ebiragala ebizimbya akanyama/ omubiri) ng'okyekuba na mpiso?<br><br>• *Indicate you are referring to non-medical use only.*	Nedda<br>Yye, naye ssi mu myezi 3 egiyise<br>Yye, mu myezi 3 egiyise
